# Oranges as Medicine

**Published:** 1893-07

**Authors:** 


### ORANGES AS A MEDICINE.

It cannot be emphasized too strongly, especially at this season of the year and in this part of the world, that pure fruit juice is one of the best blood purifiers and system-regulators there is. In fact, it is said to be the base of physicians' prescriptions in cases of depleted systems and impure blood.

There are people in this place who testify to this fact, particularly as to oranges. Some people, who have heretofore eaten fruit between meals or just before retiring and condemned it as injurious, have learned in California to eat one or two oranges with nearly every meal, particularly breakfast, and have found to their pleasant surprise that it was better than any medicine ever taken.

Many remarkable things have been claimed for oranges taken as a food, such as making the complexion clear and beautiful, curing the drink habit and numerous other things as varied and marvelous as the achievements of corn medicines, and there are, doubtless, persons who have made themselves miserable and ridiculous eating oranges by the wholesale in the endeavor to accomplish some such impossible result.

But thousands of persons can testify that a judicious use of oranges is a good thing; but a few precautions must be taken. In the first place, buy nothing but good fruit, especially ripe fruit. Green or bad fruit cannot be good for anybody. Then, if you do not eat the orange out of the shell with a spoon as many prefer to do, be sure to peel it carefully. The white pith lying beneath the yellow rind is one of the most indigestible substances known in the vegetable world. It is better to eat oranges with a spoon and take as little as possible of the cellular matter. Do not eat too many oranges at first; but if the habit of eating them with meals is once formed, a person will never be satisfied to eat a meal without fresh fruit of some kind. The habit will work wonders in a short time toward regulating the system, keeping the blood in good condition and creating a healthy appetite.

There is no doubt that half the pleasant flavor of the orange is destroyed by the difficulty of eating it gracefully, although that is a feat of which, like building an open fire, every one imagines his method is the best. So great a terror does an orange inspire in a woman at luncheon with the fatal example which has so often been told hanging over her of the man who broke an engagement when he saw his sweetheart hacking at one, that this delightful food is generally tabooed. One feels tempted to follow the example of the dear old lady who was in the habit of retiring to her room with an orange and locking the door after her But the mystery was lately solved at a luncheon, and the solution will be hailed with delight by housekeepers. The oranges were peeled and sliced and brought on the table cut up fine in punch glasses, in which there was a great deal of juice. In each glass there was cracked ice and sugar, and this delicious combination, which embodies all the delights of the fruit, with none of its disadvantages, is eaten with a spoon.

